# Cellular Immune Signal Exchange From Ischemic Stroke to Intestinal Lesions Through Brain-Gut Axis

**DOI:** 10.3389/fimmu.2022.688619

**Published:** 2022-04-01

**Authors:** Zizhao Yang, Fei Wei, Bin Zhang, Yun Luo, Xiaoyan Xing, Min Wang, Rongchang Chen, Guibo Sun, Xiaobo Sun

**Affiliations:** ^1^ Beijing Key Laboratory of Innovative Drug Discovery of Traditional Chinese Medicine (Natural Medicine) and Translational Medicine, Institute of Medicinal Plant Development, Chinese Academy of Medical Sciences and Peking Union Medical College, Beijing, China; ^2^ Program in Neuroscience and Behavioral Disorders, Duke-NUS Medical School, National University of Singapore, Singapore, Singapore

**Keywords:** ischemic stroke, brain-gut axis, intestinal complications, cellular immunity, necrotizing enterocolitis, gut microbiota dysbiosis, inflammatory bowel disease, colorectal carcinoma

## Abstract

As a vital pivot for the human circulatory system, the brain-gut axis is now being considered as an important channel for many of the small immune molecules’ transductions, including interleukins, interferons, neurotransmitters, peptides, and the chemokines penetrating the mesentery and blood brain barrier (BBB) during the development of an ischemic stroke (IS). Hypoxia-ischemia contributes to pituitary and neurofunctional disorders by interfering with the molecular signal release and communication then providing feedback to the gut. Suffering from such a disease on a long-term basis may cause the peripheral system’s homeostasis to become imbalanced, and it can also lead to multiple intestinal complications such as gut microbiota dysbiosis (GMD), inflammatory bowel disease (IBD), necrotizing enterocolitis (NEC), and even the tumorigenesis of colorectal carcinoma (CRC). Correspondingly, these complications will deteriorate the cerebral infarctions and, in patients suffering with IS, it can even ruin the brain’s immune system. This review summarized recent studies on abnormal immunological signal exchange mediated polarization subtype changes, in both macrophages and microglial cells as well as T-lymphocytes. How gut complications modulate the immune signal transduction from the brain are also elucidated and analyzed. The conclusions drawn in this review could provide guidance and novel strategies to benefit remedies for both IS and relative gut lesions from immune-prophylaxis and immunotherapy aspects.

## Introduction

**Figure 1:**
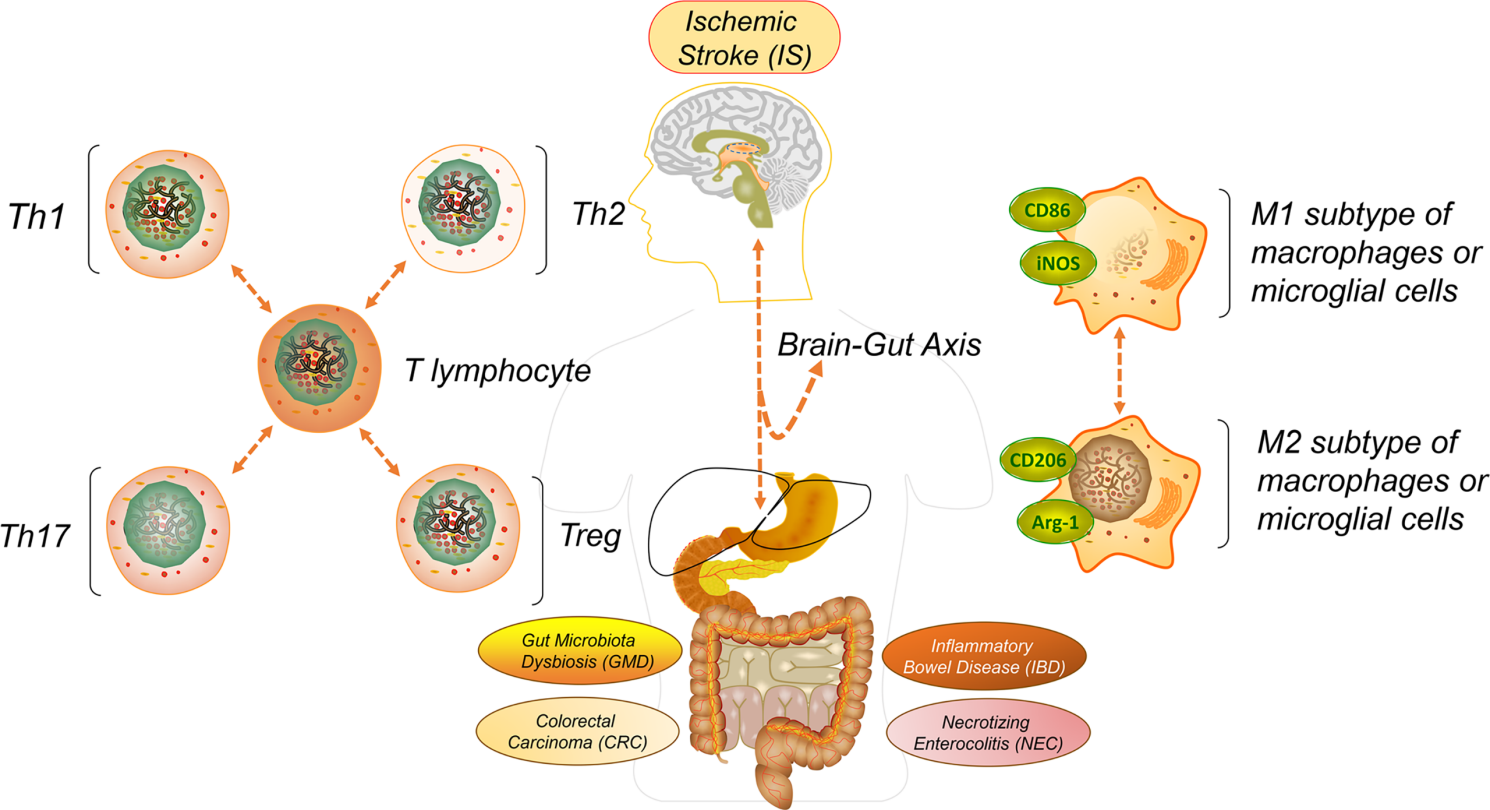
Graphical abstract. To better understand the rationale of IS initiation-induced immune signal exchange to multiple intestinal lesions or complications through the brain-gut axis, such as gut microbiota dysbiosis (GMD), inflammatory bowel disease (IBD), necrotizing enterocolitis (NEC), colorectal carcinoma (CRC), we have reviewed and summarized recent articles and drawn reasonable guidance on how to take precautions and alleviate each creation.

As one of the most high-risk cerebrum lesions in the world, ischemic stroke (IS) exhibits cerebral artery stenosis, occlusion or acute blood circulation barricade and takes possession of 75% to 85% of all-type strokes, with the specific characters of burstiness, rapidity, disability and high mortality (1–3). The pathogenesis of IS originates from multiple systematic symptoms, such as thrombus-induced atherosclerosis, cardiovascular inflammation, arrhythmia, and supplementary blood deficiency in the cerebrum (4, 5). Clinical manifestations include anterior or intracranial artery damages such as uncontrolled behavior, alogia, hypersomnia, and neurodegenerative disease, especially the onset of tumors and the growth of glioblastoma multiforme which may thoroughly destroy the brain’s central nervous system (CNS) (6–8). When a cerebral artery embolism grows, the intracellular environment experiences a conspicuous deficiency of blood, then promotes multiple immune signals such as cytokines, chemokines and matrix metalloproteinases (MMP) 1, 2 and 9 which discharges from the brain and flows into the gut (9, 10). The consequent blood reperfusion facilitates the infiltration of blood cells, including allowing natural killers (NKs), monocytes, lymphocytes or neutrophils to enter into the damaged brain parenchyma (11). During such a procedure, those signals or immune factors are transmitted into other organs, like the spleen, to separately trigger the inflammations (12). Many cases have been elucidated, but among all of the human organs the gut is the most versatile one in continually acquiring the immune signals that are released from the IS-triggered injury regions in the brain, and then conversely inducing the creation of different intestinal lesions (13–15) (**Figure 1**).

A recent study illustrated that the development of IS was corresponding to some gut abnormalities such as constipation, microbiota diversity dysbiosis, intestinal motility disorder and tract bleeding. More seriously, such abnormalities could turn into complications such as gut microbiota dysbiosis (GMD), inflammatory bowel disease (IBD), necrotizing enterocolitis (NEC) and colorectal carcinoma (CRC) (16–19). Of these, IS-mediated GMD has a relatively higher frequency of occurrence. For example, patients with IS may be diagnosed with post-stroke cognitive impairment, which reflects obvious GMD symptoms. The abundance of Firmicutes, including the same species of Clostridia and Lachnospiraceae in the guts is experiencing an obvious reduction (20). Following such phenomena, the degradation of mucus proteins and goblet T-lymphocytes apoptosis are also augmented in the intra-intestinal epithelium (21, 22). In addition, from those middle cerebral artery occlusion (MCAO) rat models, the fecal flora transplantation recreates the ecological balance of gut microbiota, alleviates the cerebrovascular embolism generation speed, then attenuates the infarct size, finally eliminates the brain edema (23). Moreover, the peripheral adaptive immunity-mediated neuroinflammatory response, which is a common stressor in IS, has a strong connection with GMD through the bidirectional hypothalamic–pituitary–adrenal axis mediated signal transmission (24). Further investigations must still be carried out to demonstrate whether microbiota dysbiosis is the prerequisite for other gut complications triggered by IS however.

T-lymphocyte colony subtypes, including helper T-cells such as Th1, Th2, Th17 and regulatory T-cells such as Treg, have different functions and performances in the beginning and during the development of IS (25). Th2 and Treg cells are antagonists in preventing inflammation in brain injuries (26), while Th1 and Th17, which are the agonists regulated by Th2 and Treg, controversially perform the neuroprotective role in the post-stroke neurogenesis process (27). Th1 secretes IL-2, IL-12, tumor necrosis factor-α (TNF-α) and interferon-γ (IFN-γ) to activate the immune response (28). Relatively, cytokines IL-6, IL-21, IL-22 and IL-17 can be secreted from Th17 (29). However, this T-lymphocyte subtype may tear down the neurovascular unit after penetrating the BBB and then work against the recovery from IS (30). Differently, Th2 and Treg, which are correlative to secreted cytokines, such as IL-4, IL-35, IL-10, transform growth factor-β (TGF-β) and exhibit anti-inflammation and protective cerebral effects (31–33). Accordingly, regulating T-lymphocyte polarization direction to Th2 and Treg contributes to IS attenuation.

The initial M2 subtype polarization of macrophage can be switched to M1. Locating on the M1 subtype of the macrophage surface membrane, CD16/32, CD86, CD40, and the major histocompatibility complex (MHC-II) could induce the discharge of proinflammatory factors including IL-1β, IL-6, IL-12, IL-23, TNFα. Some chemokines are also involved in for example like C-C motif chemokine ligand (CCL) 8, CCL15, C-X-C motif chemokine ligand (CXCL)10 and CXCL19 (34–36). Meanwhile, some transcription factors are also activated, such as STAT1, IRF3, NFκB and AP-1 (36). The M1 macrophages subtype can participate in Th1 creation, which is differentiated from CD4^+^ and CD8^+^ T-lymphocytes and plays a vital role against the formation of pathogens, and tumorigenesis (37). Correspondingly, the M2 macrophages subtype has surface membrane markers such as Arg-1, CD163, Fizz-1, scavenger receptor (SR) and mannose receptor (MR) to facilitate the releases of the chemokines like CCL13, CCL14, CCL17, CCL18, or cytokines like TGFβ, IL-10 (38, 39). Such signals will drive the decrease of inducible nitric oxide synthase (iNOS) and the increase of arginase-1 (Arg-1), which could break the Arginine metabolism’s balance, or lead to nitric oxide (NO) creation (40, 41). Differently to the M1 subtype, M2 macrophages benefit revascularization, tissue remodeling and wound healing (42–44). Interestingly, the IS-mediated polarization of microglial cells is also stimulated and reversibly differentiated from M2 subtype polarization to M1 (45). Unlike M1 and M2 macrophage polarization, the M1 microglial cells subtype could deteriorate the IS-mediated area of the brain injury, and even damage the central nervous system (46). Those hints are all manifested that the polarization in either macrophage or microglial cells should play completely opposite roles to the IS triggered intestinal lesions.

**Figure 2:**
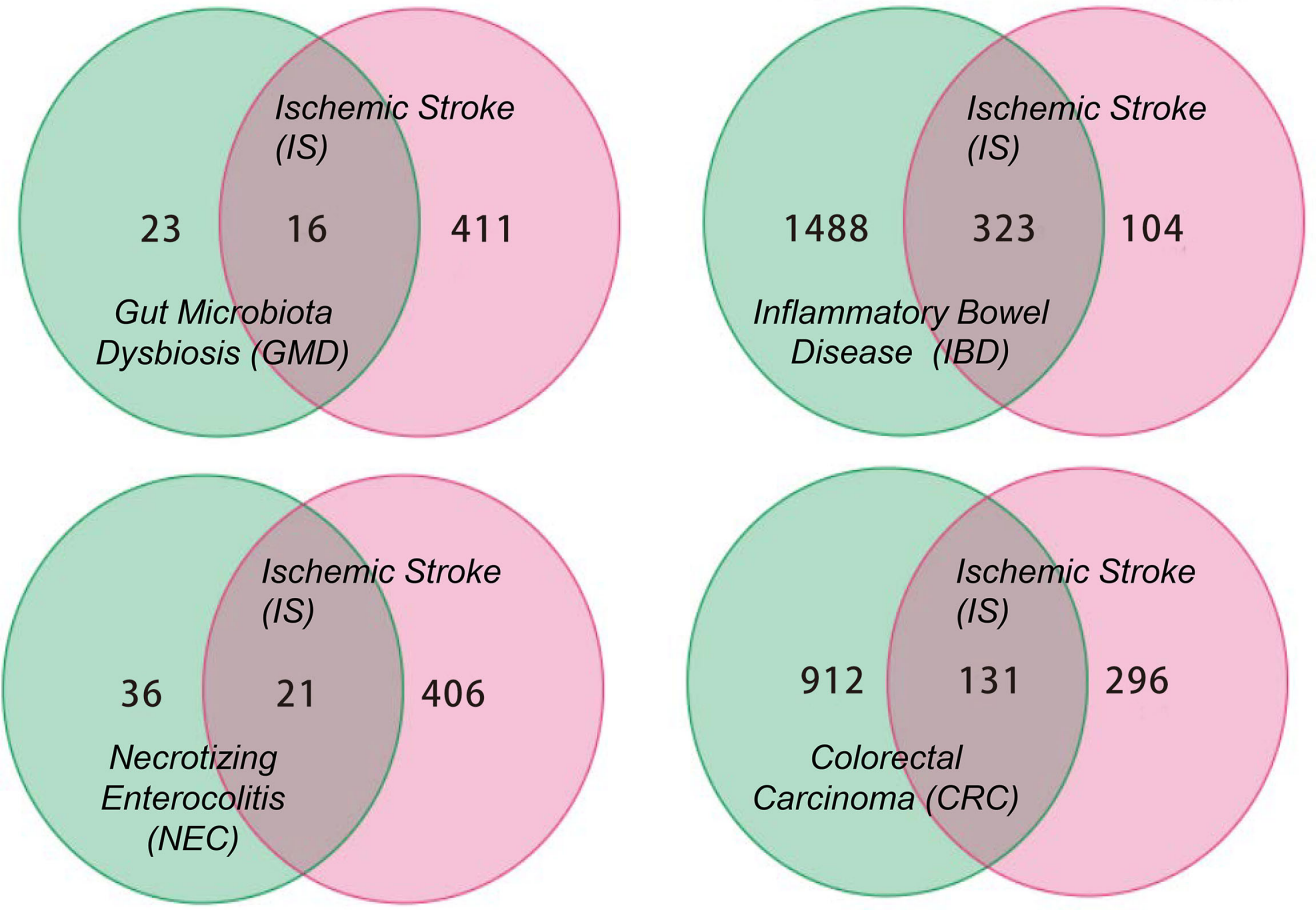
The Venn diagram for all the potential targets correlated to IS and GMD, IBD, NEC, CRC respectively. The numbers in each diagram represent the quantification statistics of the potential target numbers for IS and other lesions. All the data has come from the multiple databases of STRING, TCMSP, Swiss Target Prediction, SEA, GeneCards, DrugBank and DisGeNet and has been reproduced using Microsoft Software.

**Figure 3:**
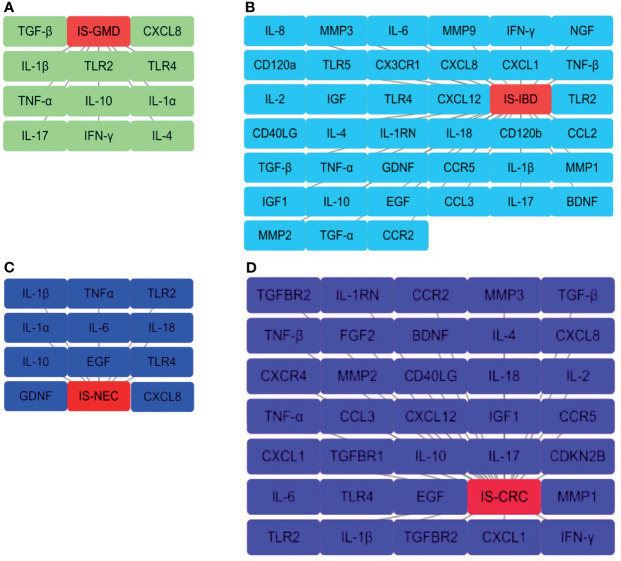
Interaction networks for the selected cellular immune signals from overlapping targets. These are those that have been identified from the overlapping results of the Venn diagram statistics in **(A)** IS-GMD, **(B)** IS-IBD, **(C)** IS-NEC, **(D)** IS-CRC group to correlate with T-lymphocytes differentiation or macrophage and microglial cells polarization. The node from each pane represents a selected target and the gray line reflects the interaction with each group. All the data has been reproduced through Cytoscape Version 3.6.0. software.

This review summarizes the involvement of IS in the immune signal exchange from the axis of the gut to the brain, as well as from the brain to the gut, when under multiple intestinal lesions. We screened all the potential independent or overlapping targets for IS, as well as each of the intestinal complications, through a target interaction network based on the STRING, TCMSP, Swiss Target Prediction, SEA, GeneCards, DrugBank and DisGeNet databases (**Figure 2** and **Supplementary Material**). After that, we selected all the cellular immune signal targets with significant differences from the patients suffered with IS, including chemokines, cytokines, interleukins, interferons, neuro-immune and hormone-immune factors. It is worth to mention that those of them are also correlated to the events of T-lymphocytes differentiation, macrophage or microglial cells polarization. Following this, we then established four cluster panels to indicate their inner connections through the Software Cytoscape Version 3.6.0 (**Figure 3**). This data and the recent reports were gathered together to highlight the rationales behind such immunotherapeutic regulations. Such of that may provide cure strategies for how to prevent IS-induced gut complications, as well as facilitate medicinal developments for these diseases.

## Immune Signal Exchange Between IS and Mediated Gut Microbiota Dysbiosis (GMD)

The presence of gut microbiota dysbiosis (GMD) will further break the immune system’s balance and stability by leading to a declining biosynthesis of γδT lymphocytes, which directly impairs the regulatory T-lymphocyte differentiation even the neurological functions and infarct size in IS (47, 48). It is worth mentioning that IL-17 secreted from γδT lymphocytes plays a crucial role in such an intervention procedure (49). Recent reports demonstrated that IS-induced GMD gave rise to the migration of T-lymphocytes from the Peyer patches, or intestinal lamina propria, to the brain leptomeninges, and then stimulated the augmentation of γδT lymphocytes in these regions of the brain (47). However, the accumulated γδT could discharge more IL-17 secretions and deteriorate brain injuries (50). Besides, Singh and colleagues transplanted the feces from the mice suffering with IS into the germ-free mice with the same disease. Then, they realized either IFN-γ or IL-17 surged in both of Th1 and Th17 cells during mice brain injuries (48). Also, they found that bacterial colonization reduced the stroke volumes in the colonized ex-germfree and specific pathogen-free (SPF) mice, when compared to germfree ones, meanwhile it increased the cerebral cytokines expression, such as IL-1β, TNF-α, IL-10, IL-17, as well as the microglia or macrophage numbers (51). Reversely, removing IL-17 leads to a provision of blood and oxygen for the brain’s recovery, IS symptom alleviation and GMD rebalance, also it activates the body’s defense function (52, 53). For example, constraining the T-lymphocyte differentiation to an excessive release of γδT and IL-17 in the mesenteric dendritic cells would restore GMD and begin to promote the accumulation of CD4^+^ T lymphocytes (Th17), thereby holding back the immigration of γδT lymphocytes from the gut to the region of the brain’s injuries (54, 55).

Except from the specific interleukin, some exogenous compounds metabolized by gut microbiota also correlate to γδT boosting under IS. For example, lactulose alters the composition of gut microbiota and contributes to the number of Foxp3+ Treg cells that markedly drop off. Those phenotypes are also responsible for the proliferation of T-lymphocytes through secreting IL-6, IL-17, IL10 and TNF-α cytokines and then up-regulating the short-chain fatty acids (SCFAs), which contain acetate, propionate and butyrate, in the intestine or serum (56). Some scientists also noticed that gut microbiota-mediated SCFAs synthesis can perform an immune-modulating effect on the microglia cells-induced synaptic plasticity recovery after the generation of IS, as well as evoke the secretion of IL-10 from T-lymphocytes-differentiated Th1 cells in the gut (57, 58). The abnormal synthesis of such fatty acids driven by IS promotes the creation of GMD and γδT and then revitalizes inflammation spreading (59). Besides, administering a single-course of amoxicillin to the mice may severely disrupt the gut microbiota’s components, enrich the pathobionts of Klebsiella and Escherichia-Shigella but dramatically delete the prebiotics of Bifidobacterium and Lactobacillus (60). Moreover, amoxicillin retards the accumulation of Treg, but facilitates the differentiation of T-lymphocytes with helper Th1 in the gut (61). Further study has also revealed that amoxicillin can be used to antagonize the Varicella Zoster or Syphilis virus, following with recovering the homeostasis of cerebrospinal fluid circulation in the brain (62, 63). All the evidence implies that the compromising of the GMD by amoxicillin is related to the immune signal circulations from the gut to the brain.

Some growth and neurotrophic factors such as the nerve growth factor (NGF), fibroblast growth factor (FGF), glial cell-derived neurotrophic factor (GDNF) or brain-derived neurotrophic factor (BDNF), can also take part in IS-induced immune signal exchanges and migration (64–66). By coordinating with other interleukins, interferons, chemokines or cytokines, BDNF can participate in the microglial cells’ polarization-induced immune signal exchange through the brain-gut axis, while also breaking the gut microbiota’s balance and setting up a responsibility for the creation of GMD (67). Downregulation of FGF, GDNF and their receptors during the initiation of IS connects with the M1 microglial cells subtype. This is followed by high volume IL-23 secretions to reverse the outgrowth of phosphatidylinositol 3-kinase (PI3K) and cAMP-response element binding protein (CREB) pathways. It is done while GMD is facilitated through augmentations of NF-κB, GSK-3β, TGF-β-mediated Smad2/3 and p38 MAPK signals (68, 69). In reverse, as an interplay communication, some pro-inflammatory cytokines such as IL-6 and TNF-α have high levels in the spinal cord, which when under GMD conditions, can accelerate the release of growth-associated protein 43 (GAP-43), neurotrophin-3 (NT-3) and BDNF from the brain neurocytes, and then modulate the axonal plasticity to spontaneously attenuate the IS-derived brain injuries (70). Similarly, the lipopolysaccharide (LPS) induced inflammatory formation impacts the constitution of the gut microbiota in aggravating GMD. This thus contributes to the discharging of BDNF from the gut’s microglial cells, followed by an increase in multiple IS inactivation cytokines excreted from astrocytes, such as CCL2, IL-15, IL-17, IL-10 and TGF-β (71–76). Interestingly, GMD-mediated abnormal synthesis of SCFAs can ameliorate microglia cells transition, intervene in the immune signal transmission induced by IS and has been confirmed to activate the pathway of BDNF-TrkB and exert neurogenesis (77, 78). This implies that interleukins, endogenous compounds and neurotrophic factors all influence the immune responses in either the brain or the gut.

## Inflammatory Bowel Disease (IBD)-Induced Immune Signal Exchange to IS

As a severe gut disorder, inflammatory bowel disease (IBD) is characterized by a recurrent and chronic gastrointestinal inflammation and it is divided into three subtypes including ulcerative colitis, Crohn’s disease and indeterminate colitis (79). The majority of clues lead to the fact that IBD initiation can be found in multiple variations for the gut and relative symptoms, such as gut permeability, microbiota colonization enlargement, bacteria translocation and hypothalamic-pituitary-adrenal axis response elimination (80). Out of all the IBD-induced gut variations, the immune response activation in gut mucosal cells is the most versatile one in triggering T-lymphocyte differentiation and transformation into helper Th1, Th2, Th17 or regulatory Tregs (81). However, only the other stress-mediated Th2 and Treg from M2 macrophages could be responsible for IBD alleviation (82). Proinflammatory Th1 and Th17 polarization, stimulation and macrophage infiltration in the gut antagonize the immunopathology of IBD (83). Similarly, T-cells’ differentiations between Th1 and Th17 expedite IS development (84), which implies that restricting such a procedure may be beneficial to either IS or IBD patients. Moreover, the migration of CX3CR1^+^ CD28^-^ CD4^+^ T-lymphocytes reflecting as a Th1 phenotype localized in the brain leptomeninges enhances IS-mediated IBD by increasing the production of multiple chemokines and the local infiltrations of cytotoxic immune cells (85). All of this elucidates the T-lymphocytes’ activities including migration, differentiation, and in particular, the polarization-triggered immune signal transmission that can influence the IS-derived onset of IBD.

Some pathogen-associated molecular patterns (PAMPs), which are associated to the pattern recognition receptors (PRRs) that mediate immune signal release and communication, are also responsible for GMD-induced IBD, and are verified to have intersections with an IS-origin (86). Current reports show that Toll-like receptors (TLRs) from PAMPs modulated the susceptibility and permeability of IBD were further able to participate in the GMD and immune signal exchange (87). The distributions of the TLR subtypes are found in multiple types of human cellular surface membranes, such as intestinal epithelial cells (IECs), macrophages, dendritic cells (DCs), mast cells, lymphocyte, neutrophils and CNS enteric glial cells (EGCs) (88–91). Therefore, studies demonstrated that microbial component variation in the gut can regulate TLR5, trigger an increase in the T-lymphocytes transformation and even cut-off the interferons and inflammation factors, including those of IL-1β, IL-6, IL-8 and TNF-α (92). One of the subtypes, TLR2, is now confirmed to be concerned with GMD-mediated aberrant immune responses, and taking that out of animals can assuage the dextran sulphate sodium salt (DSS)-induced colitis (93). Similarly, when comparing the mice, scientists found that relative to the IBD-mediated GMD group, TLR4 knockout mice displayed more neuroinflammation and gastrointestinal disturbances (93, 94). In addition, some other vital genes also involved in the aberrant immune response to GMD in IBD patients, such as nucleotide-binding oligomerization domain-containing-2 (NOD2), Caspase-recruitment domain 15 (CARD15), immunity-related GTPase M (IRGM), autophagy-related 16-like 1 (ATG16L1) (95). These independently take part in the processing of innate human immune responses and coordinate with vital pathways to modulate the extent of IS through proinflammatory Th1/Th17 polarization or macrophage infiltration (96–98). These perform a central role in immune signal amplification and transmission during the pathogenesis of IBD.

## Necrotizing Enterocolitis (NEC)-Mediated Immune Signal Exchange to IS

As one of the most devastating intestinal lesions, necrotizing enterocolitis (NEC) affects 5–10% infants, or 2-5% of all preterm neonates, however its clinical treatment in neonatology still remains unclear (99). The overall mortality rate approaches 100% in infants suffering from NEC, along with other congenital fundamental diseases such as intestinal perforation, peritonitis and so on (100). It was once considered to primarily originate from ischemic mucosal injury from the immature gut of a preterm, but now theories on its cause have turned to some major antenatal or postnatal risk factors. These primarily include chorioamnionitis and maternal antibiotics abuse (101, 102). Correspondingly, postnatal risk factors include gut motility and enzymatic function reverse, hemostasis of immune system disorder, mucus production and composition alteration, defense stress, intestinal hypoxia-ischemia-reperfusion activation, and normal neonatal gut colonization intervention (103). NEC-induced GMD is a common feature in these patients (104). For example, insufficient colonization by Firmicutes and Proteobacteria in preterm babies is attributed to immune tolerance inactivation and provoking an overwhelming intestinal inflammation reaction followed by NEC (105). Successive studies have also shown that during the development of NEC, a significant alteration existed in each fecal microbiota colonization of Klebsiella, Tatumella, Citrobacter and Sphingomonas spp. Under such conditions, the enterococcal microbe of the depletion of the gut’s microbiota will also be determined (106, 107).

Manifestations have demonstrated that the initiation of IS is tightly connected to NEC, and then it is noticeably responsible for its development. Some tools such as remote ischemic conditioning (RIC), can be used to prevent single intestinal injuries (18). Under such conditions, the polarization and anti-inflammation of the macrophage have been activated (108). Currently, the counting number ratio of M1/M2 subtypes of polarized macrophage has been unanimously approved as an important determination index and is used for measuring and verifying a patient’s degree of NEC (109, 110). During such a procedure, multiple immune signals are stimulated to play the repairing role for NEC. For instance, through intraperitoneal injections of Gr-1 antibodies combined with carrageenan with significantly depleted polymorphonuclear leukocytes (PMNs) and macrophage in the NEC mice model induced by cronobacter sakazakii (CS), led scientists to realize that the cytokines including IL-1β, IL-12, IL-6, TNF-α, iNOS were all up-regulated, the apoptosis of vascular epithelial cells significantly surged, and those with variations could exacerbate NEC-mediated intestinal lesions (111). Anand and colleagues explored how extra IFN-γ can activate the function performance of macrophages by inhibiting the phosphorylation of connexin, while Cx43 in enterocytes can then turn down the intercellular junctions and migration ability. Therefore, such interferon will alleviate the risks for macrophage M1 subtype transformation and prevent the pathogenesis of NEC (112, 113). Halpern and colleagues elucidated how rat milk substitute (RMS), asphyxia and cold stress can induce the creation of NEC in newborn rats. They also investigated how IFN-γ secretion-boosting triggered the aggregation of cytokine IL-18 and IL-12-positive monocytes or macrophages (IL-12 is the marker of M1 macrophage subtype) (114). Taken together, these findings imply that IFN-γ could be vital for macrophage polarization and M1 subtype creation during the early onset of NEC.

## Immune Signal Exchange Between Colorectal Carcinoma (CRC) and IS

Colorectal carcinoma (CRC) is one of the most common malignant carcinomas in a human being’s digestive tract and has major repercussions on the efficacy of immunotherapy. Ranking third in terms of incidence and second in its worldwide mortality rate, the surging number of CRC patients is now counted at 1.8 million new cases and 881,000 deaths in 2018 alone, and it is predicted to increase to 2.2 million new cases and 1.1 million deaths worldwide by 2030 (115, 116). Patients with IS may have a higher risk of suffering from CRC (117). Both IS and CRC share the same complications, including atrial fibrillation (AF), arterial thromboembolism, hypercoagulability induced by elevated carotid endarterectomy (CEA) and cardiovascular disease (CVD), which fully illustrates the tight physiological connection between each of them (19, 118, 119). On the other hand, GMD, which broke the immune hemostasis, is also considered responsible for the initiation and development of CRC (120). Some species such as Enterococcus faecalis, Clostridium septicum, Bacteroides fragilis, Helicobacter pylori, Streptococcus bovis, Escherichia coli and Fusobacterium spp. are supposedly vital in the pathogenesis of colorectal tumor growth and metastasis (121). Recent report referred, 13 out of the 18 genera microbiota, such as Streptococcus, experienced a significant boost in the normal colorectal tissues when compared to the carcinoma ones (122). Furthermore, mutating the adenomatous polyposis coli (APC) tumor-suppressor gene in the C57BL/6J mice destroyed the diversity of the gut microbiota and spontaneously developed intestinal tumorigenesis (123). Further studies also demonstrated that as the key metabolites, overcharging of the SCFAs modulated T-cell differentiation, and in addition, not only did it deteriorate the creation of IS-induced dysbacteriosis but it was also responsible for the tumorigenesis of CRC (124–126). By suppressing the creation and release of inflammatory factors, such an aberrant phenotype will barricade the proliferation of the effector CD4^+^ T-lymphocytes, while also breaking the intestinal immune homeostasis (127). Among all the ingredients of SCFAs, butyrate and propionate are correlated to the extrathymic generation of colitis and functional differentiation and proliferation elimination of regulatory T-lymphocytes (128–130). Moreover, both of them can influence the modulation of the gut’s macrophages polarization by suppressing the histone deacetylase which thereby drives the tolerance increase for intestinal dysbacteriosis (131). Accordingly, GMD-induced CRC impairs the gut-brain signal circulation and mainly dominates through endogenous substances, metabolites and substitutions.

During the commencement of IS, the majority of immunosuppressive molecules such as the immune checkpoint receptor of programmed cell death 1 (PD-1) or programmed cell death ligand 1 (PD-L1) are stimulated and activated to prevent inflammatory generations such as the arterial wall damage by vasculitis or the mediated multiple immune responses (132). Reversely, PD-L1 deficiency amplifies the infarct sizes and deteriorates neurological deficits in the MACO C57BL/6 mice, and then promotes the polarization of macrophages or microglial cells (133). High volumes of IFN-γ conversely stimulate the up-regulation of PD-L1 and then mediate the multiple immune suppression for IS correlative cytokines or signal amplification pathways, such as PI3K/AKT and JAK/STAT3, to attenuate the anti-tumor immune positive responses (134, 135). Accordingly, PD-1 or the ligand PD-L1 is crucial for either IS or the tumorigenesis procedure of CRC (136, 137). In the early stages of CRC, characterized mismatch repair-deficient (dMMR) and microsatellite instability (MSI) have a relatively high level of CD8^+^ cytotoxic T-cells as well as PD-1/PD-L1 expression (138, 139). Further reports demonstrated that PD-1/PD-L1 can regulate the Th9 tumor-infiltrating lymphocytes (TILs) in CRC and drive the CD8^+^ T-cell expansion but not CD4^+^ T-cell (140). Interestingly, CD163^+^ M2 macrophages accumulation significantly increased in the cerebral infarction area of those patients suffering with IS (141). To strengthen the augment, PD-L1 was found to be expressed on tumor cells or CD68^+^/CD163^+^ M2 tumor-associated macrophages in MSI CRC patients and was attributed to tumor invasion extension and immune escape (142). Since polarized macrophages can be infiltrated from the brain to the gut with CD4^+^ T-lymphocyte (143), it may be possible that PD-L1 is also involved with such a macrophage transformation. Further investigations are necessary in order to research whether the activation of PD-1 and PD-L1 under CRC induces the polarizations of macrophages or whether microglial cells can transmit the immune signals back to the brain. Once this is elucidated, we may be able to completely understand the integral picture on how to intervene with CRC or IS through PD-1 or PD-L1 blockers.

## Conclusion and Future Perspective

As one of the major lethal diseases in the world, scientists are primarily focusing on IS’ pathogenesis mechanisms but overlooking the potential complications, especially potential lesions in the gut, such as GMD, IBD, NEC and CRC. Through the brain-gut circulation, IS-induced signal exchanges coordinate with events including the activation of the neurohormone secretion, immunosignal release, lymphocyte differentiation, and even the oncogene resuscitation. It is worth mentioning that these major intestinal lesions accompanied with IS should be independent and mutually reflected in one or more complications. Recent reports reflected that the mice accepted the fecal transplants from higher stroke-associated dysbiosis patterns and that patients could suffer from severe brain injuries when accelerating the IL-17^+^ γδ T-cell creation in their guts (144). Relatively, the increase in expression of IL-17^+^ γδ T-lymphocytes are certified as the main elements that induce IBD, NEC and CRC (145–147). Accordingly, analyzing the functional alterations for specific gut microbiota colonization would be helpful to understand which species of flora are the most probably related to such phenomena.

The secreted interleukins from the aberrant differentiations or polarizations of T-lymphocytes, as well as the macrophages or microglial cells, form up the major communication signals for IS-derived intestinal complications. For example, the majority of reports implied that IL-6 was the most vital biomarker in IBD or NEC initiation (148, 149). Coincidentally, this interleukin is also the predictive biomarker for infections associated with strokes, and those situations could significantly increase its secretion (150). Moreover, IFN-γ could be another predominant transmissive immune signal related to gut complications and IS. Following IS deterioration, there is a boost in IFN-γ secretions (151). More IFN-γ triggers the generation of Th1 and Th17, which in parallel will stimulate PD-L1 recruitment and the JAK/STAT pathway, that in turn is relative and conducive to a CRC prognosis (152, 153). Further study still needs to be carried out in order to verify whether eliminating IFN-γ production could alleviate IS or CRC, or even reverse T-lymphocyte polarization.

In summary, this review illustrates cellular immunity-induced signal exchanges from IS-derived brain-to-gut complications including GMD, IBD, NEC and CRC. Some important cytokines and interleukins could become the primary therapeutic targets used to intervene in these complications, though basic scientific studies and clinical trials still need to be performed to verify the inner rationales of their roles in IS. The synergistic interaction between IS and intestinal complications would be particularly valuable for the efforts to modulate immunity for therapeutic purposes, as well as to advance stroke therapy to the next level.

## Author Contributions

ZY has drafted this manuscript. FW, BZ, and XX have provided the editing and writing assistance and suggestions. YL, MW, and RC have critically revised it for important intellectual content. GS and XS have approved the final version for publication. All authors contributed to the article and approved the submitted version.

## Funding

This work was supported by the Natural Sciences Foundation for Young Scientists of China (82104494), key project grant from the National Natural Science Foundation of China (U20A20405), National Natural Science Foundation of China (81891012) and CAMS Innovation Fund for Medical Sciences (CIFMS) (2021-12M-1-031).

## Conflict of Interest

The authors declare that the research was conducted in the absence of any commercial or financial relationships that could be construed as a potential conflict of interest.

## Publisher’s Note

All claims expressed in this article are solely those of the authors and do not necessarily represent those of their affiliated organizations, or those of the publisher, the editors and the reviewers. Any product that may be evaluated in this article, or claim that may be made by its manufacturer, is not guaranteed or endorsed by the publisher.

## Supplementary Material

Supplementary Data Sheet The statistics of all the potential varied targets corelated to IS combing with each complication of GMD, IBD, NEC, CRC, respectively. All the data has come from the multiple databases of STRING, TCMSP, Swiss Target Prediction, SEA, GeneCards, DrugBank and DisGeNet and has been reproduced using Microsoft Software.
